# Clinical, socioeconomic, and behavioural factors at age 50 years and risk of cardiometabolic multimorbidity and mortality: A cohort study

**DOI:** 10.1371/journal.pmed.1002571

**Published:** 2018-05-21

**Authors:** Archana Singh-Manoux, Aurore Fayosse, Séverine Sabia, Adam Tabak, Martin Shipley, Aline Dugravot, Mika Kivimäki

**Affiliations:** 1 INSERM, U1018, Centre for Research in Epidemiology and Population Health, Hôpital Paul Brousse, Villejuif, France; 2 Department of Epidemiology and Public Health, University College London, London, United Kingdom; University of Oxford, UNITED KINGDOM

## Abstract

**Background:**

Multimorbidity is increasingly common and is associated with adverse health outcomes, highlighting the need to broaden the single-disease framework that dominates medical research. We examined the role of midlife clinical characteristics, socioeconomic position, and behavioural factors in the development of cardiometabolic multimorbidity (at least 2 of diabetes, coronary heart disease, and stroke), along with how these factors modify risk of mortality.

**Methods and findings:**

Data on 8,270 men and women were drawn from the Whitehall II cohort study, with mean follow-up of 23.7 years (1985 to 2017). Three sets of risk factors were assessed at age 50 years, each on a 5-point scale: clinical profile (hypertension, hypercholesterolemia, overweight/obesity, family history of cardiometabolic disease), occupational position, and behavioural factors (smoking, alcohol consumption, diet, physical activity). The outcomes examined were cardiometabolic disease (diabetes, coronary heart disease, stroke), cardiometabolic multimorbidity, and mortality. We used multi-state models to examine the role of risk factors in 5 components of the cardiometabolic disease trajectory: from healthy state to first cardiometabolic disease, from first cardiometabolic disease to cardiometabolic multimorbidity, from healthy state to death, from first cardiometabolic disease to death, and from cardiometabolic multimorbidity to death. A total of 2,501 participants developed 1 of the 3 cardiometabolic diseases, 511 developed cardiometabolic multimorbidity, and 1,406 died. When behavioural and clinical risk factors were considered individually, only smoking was associated with all five transitions. In a model containing all 3 risk factor scales, midlife clinical profile was the strongest predictor of first cardiometabolic disease (hazard ratio for the least versus most favourable profile: 3.74; 95% CI: 3.14–4.45) among disease-free participants. Among participants with 1 cardiometabolic disease, adverse midlife socioeconomic (1.54; 95% CI: 1.10–2.15) and behavioural factors (2.00; 95% CI: 1.40–2.85), but not clinical characteristics, were associated with progression to cardiometabolic multimorbidity. Only midlife behavioural factors predicted mortality among participants with cardiometabolic disease (2.12; 95% CI: 1.41–3.18) or cardiometabolic multimorbidity (3.47; 95% CI: 1.81–6.66). A limitation is that the study was not large enough to estimate transitions between each disease and subsequent outcomes and between all possible pairs of diseases.

**Conclusions:**

The importance of specific midlife factors in disease progression, from disease-free state to single disease, multimorbidity, and death, varies depending on the disease stage. While clinical risk factors at age 50 determine the risk of incident cardiometabolic disease in a disease-free population, midlife socioeconomic and behavioural factors are stronger predictors of progression to multimorbidity and mortality in people with cardiometabolic disease.

## Abstract 2

Archana Singh-Manoux and colleagues report on the contribution that midlife socioeconomic and behavioural factors make to multimorbidity and mortality in those with cardiometabolic disease.

## Author summary

**Why was this study done?:**

- The prevalence of cardiometabolic multimorbidity increases with age, and any combination of diabetes, stroke, and coronary heart disease is associated with multiplicative mortality risk.
- Previous studies have examined either risk factors for multimorbidity or the manner in which multimorbidity shapes adverse health outcomes. No previous study to our knowledge has examined how socioeconomic, behavioural, and clinical risk factors shape the development, progression, and outcome of cardiometabolic multimorbidity.

**What did the researchers do and find?:**

- Data were collected on socioeconomic, behavioural, and clinical risk factors at age 50 years on 8,270 participants from the Whitehall II study, and the participants were followed over a mean 23.7 years for incident cardiometabolic disease (diabetes, coronary heart disease, or stroke), cardiometabolic multimorbidity (2 or more cardiometabolic diseases), and mortality.
- Clinical risk factors (hypertension, overweight and obesity, high cholesterol, and family history of diabetes or cardiovascular disease) were important predictors of first cardiometabolic disease. However, socioeconomic and behavioural factors (physical activity, alcohol consumption, diet, and smoking) determined progression to multimorbidity, and only behavioural risk factors predicted mortality among participants with cardiometabolic disease or cardiometabolic multimorbidity.
- When risk factors were considered individually, smoking was associated with accelerated transitions in the trajectory from the development of a first cardiometabolic disease to multimorbidity and death.

**What do these findings mean?:**

- By considering risk factors in the progression from a disease-free state to death, we determined the changing influence of socioeconomic, behavioural, and clinical risk factors.
- Our findings demonstrate that a simple focus on one point in the health trajectory of individuals misses the changing role of risk factors in the development, progression, and outcome of cardiometabolic multimorbidity, a major public health challenge worldwide.

## Introduction

Multimorbidity refers to the co-occurrence of multiple chronic conditions in the same person [1], and is known to increase with age [2–11]. Multimorbidity is associated with poor quality of life, higher healthcare costs, and greater risk of disability and mortality [11,12]; the effects of multimorbidity on adverse outcomes for the patient and the healthcare system are greater than might be expected from chronic conditions occurring on their own [11,13–15]. A recent study on cardiometabolic multimorbidity, defined using diabetes, coronary heart disease (CHD), and stroke, found each additional disease to double the risk of death [4].

Studies on risk factors for multimorbidity have shown the importance of clinical risk factors [16,17], socioeconomic factors [3,8,16,18], and behavioural factors [16,19,20], but it remains unclear whether these factors affect the risk of developing a first chronic condition or progression to multimorbidity. In addition, although prognostic studies show greater risk of adverse outcomes in those with multimorbidity, the factors that modify this risk remain unknown. A further limitation of previous research is the piecemeal approach, where studies examine the risk factors for multimorbidity and the prognosis of multimorbidity in separate analyses, often with cross-sectional data used to examine the former. Thus, the characteristics that adversely affect temporal progression from a first chronic disease to multimorbidity and subsequent mortality are unknown. The identification of such factors would pave the way for interventions that target the specific factors associated with the incidence and prognosis of multimorbidity.

In order to address some of these limitations, we examined the role of clinical, socioeconomic, and behavioural factors at age 50 years in the transitions from a healthy state to first cardiometabolic disease, cardiometabolic multimorbidity, and subsequent mortality using data spanning 30 years. The main focus of our analyses was the relative importance of clusters of clinical, social, and behavioural risk factors; however, we also examined the associations of individual behavioural and clinical risk factors with transitions.

## Methods

### Study population

Participants were drawn from the Whitehall II study, an ongoing cohort study of 10,308 persons (6,895 men and 3,413 women), aged 35–55 years at study recruitment in 1985 [21]. At baseline, participants responded to a questionnaire and underwent a structured clinical evaluation consisting of measures of anthropometry, cardiovascular and metabolic risk factors, and diseases. Follow-up clinical assessments have taken place approximately every 4–5 years (1991, 1997, 2002, 2007, 2012, and 2015), with every wave taking approximately 2 years to complete. Informed, written consent from participants and research ethics approvals (University College London [UCL] ethics committee) are renewed at each contact; the latest approval was by the Joint UCL/UCLH Committee on the Ethics of Human Research (Committee Alpha), reference number 85/0938.

### Assessment of risk factors

We extracted data on risk factors at the clinical examination closest to age 50 years, allowing a ±5-year margin, to remove the effect of baseline age in our estimates. This was done by using data from the first 4 waves of the study (in 1985, 1991, 1997, and 2002) when the age range of participants was 35–55, 40–64, 45–69, and 50–74 years. Thus, follow-up started at age 50 for all participants, and analyses were restricted to those free of cardiometabolic disease (diabetes, CHD, stroke) at age 50. Three sets of risk factors were examined, each on a 5-point scale, with 0 denoting the lowest risk, and 4 the highest risk.

Clinical profile was assessed using 4 measures, with thresholds defined using World Health Organization guidelines. Hypertension was defined as systolic blood pressure ≥ 140 mm Hg, diastolic blood pressure ≥ 90 mm Hg, or use of antihypertensive medication. Blood pressure was assessed as part of the clinical examination as the mean of 2 measurements taken using a Hawksley random zero sphygmomanometer with the participant in the sitting position after 5 minutes of rest. Overweight/obesity was defined as BMI ≥ 25 kg/m^2^; weight was measured in underwear to the nearest 0.1 kg on Soehnle electronic scales with digital readout (Leifheit, Nassau, Germany), and height was measured in bare feet to the nearest 1 mm using a stadiometer with the participant standing erect with the head in the Frankfurt plane. Hypercholesterolemia was defined as total cholesterol ≥ 5 mmol/l. Venous blood samples were taken after at least 5 hours of fasting, and serum obtained after centrifugation was refrigerated at 4°C and assayed within 72 hours of the blood draw. Total cholesterol was measured using a Cobas Fara centrifugal analyzer (Roche Diagnostics, Nutley, NJ). Finally, family history (parent or sibling) of diabetes and cardiovascular disease was reported as part of the study questionnaire.

The socioeconomic marker for the main analysis was occupational position, which in our study is the British Civil Service grade of employment—a comprehensive measure that reflects education, occupational status, and income, composed of a 5-level variable [21]. In sensitivity analyses we replaced occupational position with education, measured as the highest qualification on leaving full-time education (no academic qualifications, lower secondary school, higher secondary school, university, higher degree).

Behavioural factors considered were smoking, alcohol consumption, fruit and vegetable consumption, and physical activity. Each behaviour was scored as unhealthy (1 point) if recommended targets were not achieved and, in the case of dietary behaviour, to reflect previous categorisation of this measure [22]; the scores were summed to yield an unhealthy behaviour 5-point scale. The criteria for classifying participants as not achieving recommended behavioural targets were as follows: current smoking, alcohol abstention (<1 unit/week) or heavy alcohol consumption (>21 alcohol units/week in men and >14 alcohol units/week in women), poor diet (fruit and vegetable consumption < 1 serving/day), being physically inactive (<2.5 hours/week of moderate or vigorous physical activity) [23].

Covariates included age, sex, race (white, non-white), marital status (single, non-single), and birth cohort (4 categories: ≤1935, 1936–1940, 1941–1945, >1945).

### Ascertainment of cardiometabolic diseases and multimorbidity

Incidence of cardiometabolic multimorbidity [4], defined as at least 2 of CHD, stroke, and diabetes, was based on data from the clinical assessments between 1985 and 2016 and linkage (until 31 August 2017) to the national Hospital Episode Statistics (HES) database with in- and outpatient data, undertaken using the National Health Service (NHS) identification number. In the UK, the NHS provides most of the healthcare; private medical insurance is held by around 12% of the population (1997 figures) [24] and is used mainly for elective surgery rather than chronic conditions. The HES database contains the exact date of diagnosis (hospitalisation/outpatient visit), allowing greater precision in the time of follow-up.

Whitehall II ascertained non-fatal CHD based on 12-lead resting ECG recordings, coded using the Minnesota system, and on self-reported CHD that was corroborated with information from the general practitioner or manual retrieval of hospital records. The ascertainment included non-fatal myocardial infarction, definite angina, reported coronary artery bypass grafting, and percutaneous transluminal coronary angioplasty. HES ascertainment was based on in- or outpatient hospital consultations with ICD-9 codes 410–414, ICD-10 codes I20–I25, or procedures K40–K49, K50, K75, U19 (Office of Population Censuses and Surveys Classification of Interventions and Procedures codes for all intra-hospital medical and surgical procedures for treatment of CHD).

Stroke cases were defined using ICD-9 codes 430, 431, 434, or 436 or ICD-10 codes I60–I64 from HES records and self-reported stroke, which was validated against medical records [25].

Diagnosis of type 2 diabetes was based on the following diagnostic criteria: having fasting glucose ≥ 7.0 mmol/l (126 mg/dl) or 2-hour plasma glucose ≥ 11.1 mmol/l (200 mg/dl), reported physician-diagnosed diabetes, use of diabetes medication, or HES record of diabetes (ICD-9 code 250 or ICD-10 code E11).

### Mortality follow-up

Mortality data until 31 August 2017 were drawn from the British national mortality register (NHS Central Register). The tracing exercise was carried out using the NHS identification number of each participant.

### Statistical analysis

To allow comparisons with other findings and demonstrate the value of our analytic approach, we first used Cox regression to examine the association of risk factors (occupation, behavioural factors, and clinical profile) with incidence of first cardiometabolic disease (any of the 3), multimorbidity, and mortality. Participants were followed starting at age 50 years until the record of death or 31 August 2017, whichever came first.

All risk factor scales were on a 5-point scale where they were first treated as categorical variables (0, 1, 2, 3, or 4, where 0 indicates no risk factors/lowest risk category and 4 indicates all 4 risk factors/highest risk category). As none of the associations showed evidence of departure from linearity, we entered the risk factor scales as a continuous 5-level variable to assess the impact of clustering of risk factors. The reported hazard corresponds to the increased risk of the outcome (first cardiometabolic disease, multimorbidity, or mortality; separate analyses) in those with the highest (score of 4, the maximum score) compared to the lowest risk (score on scale = 0). The proportional hazard assumptions for Cox regression models, tested using Schoenfeld residuals, were found not to be violated.

**Fig 1:**
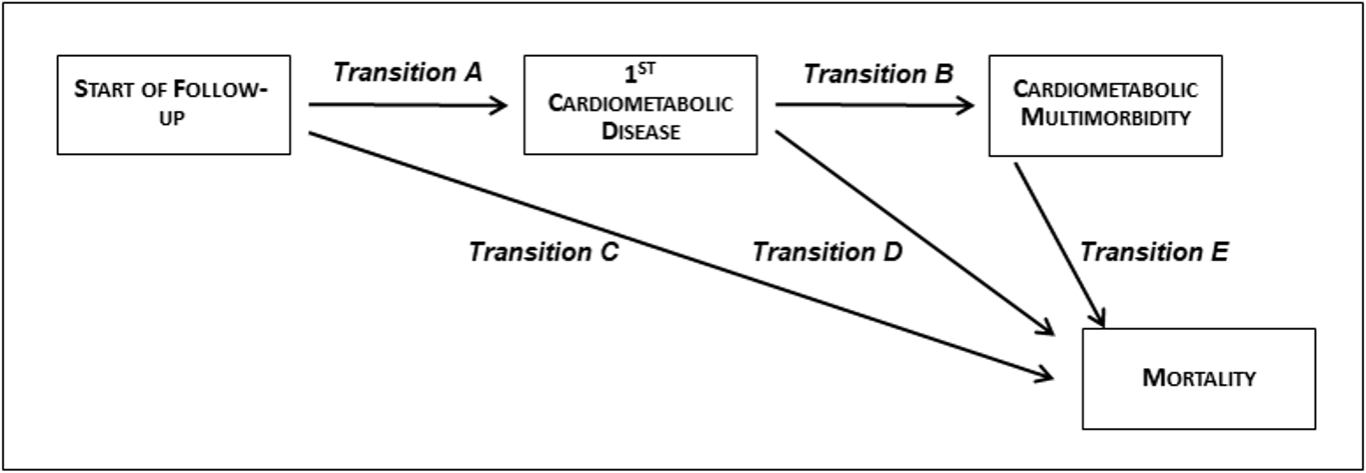
Schematic representation of the transitions between start of follow-up (healthy), first cardiometabolic disease, cardiometabolic multimorbidity, and mortality.

In subsequent analyses, we examined the role of the 3 risk factor scales (socioeconomic, behavioural, and clinical) at age 50 in the temporal disease progression from being free of cardiometabolic disease to first cardiometabolic disease, cardiometabolic multimorbidity, and death. These analyses were carried out using unidirectional multi-state models (MSMs) with Markov proportional hazards [26,27]. These models are an extension of competing risks survival analysis, allowing simultaneous estimation of the role of risk factors (here socioeconomic, behavioural, and clinical profile scales) in the transitions (for schematic representation see Fig 1) from (A) healthy to first cardiometabolic disease, (B) first cardiometabolic disease to cardiometabolic multimorbidity, (C) healthy state to death, (D) first cardiometabolic disease to death, and (E) cardiometabolic multimorbidity to death. The focus on these 5 transitions rather than all possible transitions in health states was pre-planned; an extract from our funding application can be found in S1 Protocol.

We also report the associations of the individual risk factors included in the behavioural and clinical scales in both the Cox regression models and MSMs. In all the analyses, age was used as the timescale, and analyses were additionally adjusted for sex, race, marital status, and birth cohort (Model 1). The mutually adjusted models (Model 2) contained all covariates in Model 1 and all risk factor scales (socioeconomic, behavioural, and clinical). Sex differences were tested using an interaction term between the risk factor scale and sex for both the Cox regression and the MSM analysis.

We repeated all the analyses with education replacing occupational position as a marker of socioeconomic position, as education is a more widely available measure of socioeconomic circumstances in most studies. The MSMs were run using the “mstate” package of R software; all other analyses used Stata version 14. A 2-sided *p*-value < 0.05 was considered statistically significant. This study is reported as per STROBE guidelines (S1 Checklist).

## Results

The analyses were based on 8,270 of the 10,308 participants recruited to the study in 1985 (flowchart in S1 Fig), followed over a mean 23.7 (SD = 5.9) years. As all risk factors were drawn at around age 50 years, participants older than this at baseline, those who dropped out of the study before reaching this age, and those with missing data on risk factors at age 50 were excluded from the analysis (*N* = 1,581). Table 1 presents the sample characteristics at age 50 as a function of cardiometabolic multimorbidity and mortality status at the end of follow-up; these characteristics in relation to the 3 individual diseases considered in the analyses (diabetes, CHD, and stroke) are shown in S1 Table.

**Table 1:** Characteristics of the study population at age 50 as a function of multimorbidity and mortality status at the end of follow-up (*N* = 8,270).

Characteristic	Cardiometabolic multimorbidity			Mortality		
	No	Yes	*p*-Value	No	Yes	*p*-Value
*N *	7,759	511		6,864	1,406	
Age, mean (SD)	50.2 (2.4)	51.0 (2.3)	<0.001	50.0 (2.3)	51.2 (2.5)	<0.001
Male	67.0	68.9	0.38	67.7	64.2	0.01
Non-white	9.2	25.8	<0.001	9.9	11.7	0.05
Single	24.5	22.3	0.26	23.7	27.9	0.001
No academic qualification	10.3	15.3	<0.001	10.1	12.9	<0.001
Low occupational position (clerical/support staff)	18.7	29.8	<0.001	17.7	27.7	<0.001
Physically inactive^**a**^	50.1	55.4	0.02	50.0	52.5	0.09
Poor diet^b^	37.5	44.8	0.001	36.4	45.2	<0.001
Alcohol abstention or heavy alcohol consumption^c^	36.8	42.9	0.006	36.5	40.5	0.005
Current smoker	14.9	24.7	<0.001	12.9	28.6	<0.001
Hypertension^d^	20.3	30.3	<0.001	19.1	29.5	<0.001
Overweight/obesity^e^	47.5	61.6	<0.001	47.8	51.2	0.02
Total cholesterol ≥ 5 mmol/l	86.6	90.6	0.01	86.7	87.8	0.27
Family history of diabetes or CVD	17.9	19.4	0.41	18.4	16.4	0.09

Data are percentages unless otherwise indicated.

^a^Corresponds to <2.5 hours/week of moderate or vigorous physical activity (recommended level).

^b^Corresponds to fruit and vegetable consumption < 1 serving/day.

^c^Heavy alcohol consumption was defined as >14 units/week in women and >21 units/week in men, and alcohol abstention was defined as <1 unit/week.

^d^Blood pressure ≥ 140/90 mm Hg or use of antihypertensive medication.

^e^BMI ≥ 25 kg/m^2^.

CVD, cardiovascular disease.

A total of 2,501 (30.2%) of the 8,270 participants included in the analyses experienced 1 of the 3 cardiometabolic diseases, 511 participants met the criteria of multimorbidity, and 1,406 participants died over the follow-up period, with the mean age (SD) at death being 70.0 (8.6) years. Both first cardiometabolic disease (hazard ratio [HR]: 1.81; 95% CI: 1.61–2.03; mean [SD] follow-up 10.8 [7.4] years) and cardiometabolic multimorbidity (HR: 2.36; 95% CI: 1.97–2.81; mean [SD] follow-up 6.9 [5.8] years), treated as time-varying covariates in Cox regression analysis adjusted for age, sex, race, marital status, and birth cohort, were associated with increased risk of mortality over the follow-up period (S2 Table).

In Cox regression, all individual behavioural and clinical risk factors were associated with first cardiometabolic disease (S3 Table). Overweight/obesity had the strongest association with first cardiometabolic disease, and smoking the strongest association with both cardiometabolic multimorbidity and mortality. Table 2 shows the gradient in the association between each risk score and the outcomes, justifying the use of a linear scale to assess risk in those at highest compared to lowest risk. All 3 risk scales (clinical, socioeconomic, and behavioural) at age 50 had a robust association with incidence of a first cardiometabolic disease, cardiometabolic multimorbidity, and mortality in analysis using Cox regression (Table 2); men and women were combined as there was no clear evidence of systematic sex differences (interaction terms in the footnotes of Table 2). For morbidity outcomes, the strongest associations were seen with the clinical profile scale for both first cardiometabolic disease (HR: 3.74; 95% CI: 3.14–4.46) and cardiometabolic multimorbidity (HR: 3.68; 95% CI: 2.49–5.43), with the magnitude of these associations, which estimate excess risk in those with highest compared to lowest score on the risk factor score, being very similar. The association of occupation and behavioural factors with incidence of multimorbidity was stronger than that with first cardiometabolic disease: HR of 2.43 versus 1.57 for the occupational scale and 3.02 versus 1.60 for the behavioural scale.

**Table 2:** Cox regression to assess associations of occupation, behavioural factors, and clinical profile with cardiometabolic disease, multimorbidity, and mortality.

Risk factor	*N* total	First cardiometabolic disease		Cardiometabolic multimorbidity		Mortality	
		*N* events	HR (95% CI)	*N* events	HR (95% CI)	*N* events	HR (95% CI)
**Occupational position**							
0 (high)	1,410	380	1.00 [Ref.]	53	1.00 [Ref.]	197	1.00 [Ref.]
1	1,695	448	1.05 (0.92, 1.21)	66	1.16 (0.81, 1.67)	234	1.11 (0.92, 1.35)
2	2,438	701	1.19 (1.05, 1.35)	152	1.82 (1.33, 2.50)	357	1.17 (0.98, 1.40)
3	1,122	380	1.39 (1.19, 1.61)	88	1.98 (1.37, 2.84)	229	1.55 (1.27, 1.91)
4 (low)	1,605	592	1.54 (1.32, 1.79)	152	2.38 (1.66, 3.41)	389	1.65 (1.35, 2.02)
**Occupation scale**^**a**^^**,**^^**b**^	8,270	2,501	1.57 (1.36, 1.81)	511	2.43 (1.75, 3.37)	1,406	1.68 (1.39, 2.03)
**Behavioural factors**							
0 (healthiest)	1,587	452	1.00 [Ref.]	67	1.00 [Ref.]	189	1.00 [Ref.]
1	3,063	857	1.00 (0.89, 1.12)	161	1.26 (0.95, 1.68)	453	1.30 (1.09, 1.54)
2	2,433	772	1.17 (1.04, 1.31)	171	1.66 (1.24, 2.21)	460	1.69 (1.43, 2.01)
3	1,007	355	1.37 (1.19, 1.58)	94	2.23 (1.62, 3.07)	245	2.24 (1.85, 2.72)
4 (unhealthiest)	180	65	1.64 (1.26, 2.13)	18	3.09 (1.83, 5.22)	59	3.64 (2.71, 4.89)
**Behavioural scale**^**a**^^**,**^^**c**^	8,270	2,501	1.60 (1.37, 1.88)	511	3.02 (2.13, 4.29)	1,406	3.16 (2.57, 3.90)
**Clinical profile**							
0 (healthiest)	490	91	1.00 [Ref.]	15	1.00 [Ref.]	65	1.00 [Ref.]
1	2,961	672	1.14 (0.92, 1.43)	124	1.20 (0.70, 2.05)	457	1.02 (0.79, 1.33)
2	3,201	1,051	1.78 (1.44, 2.20)	221	1.94 (1.15, 3.28)	550	1.16 (0.90, 1.50)
3	1,431	605	2.44 (1.95, 3.04)	138	2.67 (1.56, 4.55)	294	1.35 (1.03, 1.76)
4 (unhealthiest)	187	82	2.63 (1.95, 3.54)	13	2.07 (0.98, 4.36)	40	1.56 (1.05, 2.32)
**Clinical profile scale**^**a**^^**,**^^**d**^	8,270	2,501	3.74 (3.14, 4.46)	511	3.68 (2.49, 5.43)	1,406	1.65 (1.30, 2.09)

^a^HR for highest versus lowest in the scale. Analysis adjusted for age, sex, race, marital status, and birth cohort.

^b^Interaction term for sex differences: first cardiometabolic disease, *p =* 0.002; multimorbidity, *p =* 0.25; mortality, *p =* 0.04.

^c^Interaction term for sex differences: first cardiometabolic disease, *p =* 0.25; multimorbidity, *p =* 0.34; mortality, *p =* 0.11.

^d^Interaction term for sex differences: first cardiometabolic disease, *p =* 0.16; multimorbidity, *p =* 0.35; mortality, *p =* 0.07.

HR, hazard ratio.

Table 2 also shows the associations of the risk factors with mortality using Cox regression. Of the 3 risk factors, behavioural factors had the strongest association with mortality (HR of 3.16 in those with all adverse behaviours compared to those with none; 95% CI: 2.57–3.90), although both occupation and clinical profile were also associated with mortality. As with first cardiometabolic disease and multimorbidity, the linearity of associations underpins the use of the risk scale to compare mortality risk in those with the highest compared to the lowest risk. Mutual adjustment of risk factors (S4 Table) did not substantially alter associations with morbidity or mortality.

Subsequent analyses estimated the role of risk factors in the transitions shown in Fig 1. We first examined associations of individual behavioural and clinical risk factors with the transitions, which showed smoking to be the only risk factor that was important for all 5 transitions (Table 3). Subsequent analyses focused on risk factor scales; Table 4 shows the results of these analyses, and when all 3 risk factor scales (Model 2) were considered simultaneously, clinical profile had the strongest association (HR of 3.74 in those with all 4 risk factors compared to none; 95% CI: 3.14–4.45) with the incidence of first disease (diabetes, CHD, or stroke) (transition A). However, in those with a first cardiometabolic disease, it was not associated with increased risk of multimorbidity (transition B; HR: 1.29; 95% CI: 0.86–1.94). Both occupational disadvantage and adverse behavioural factors were associated with increased hazard of multimorbidity in those with a first cardiometabolic disease (transition B). Within this framework that modelled all transitions simultaneously, behavioural factors had the strongest association with prognostic transitions, i.e., transitions leading to death. This was true for transitions to death in participants without cardiometabolic diseases (transition C; HR: 2.87; 95% CI: 2.19–3.77), those with 1 cardiometabolic disease (transition D; HR: 2.12; 95% CI: 1.41–3.18), and those with multimorbidity (transition E; HR: 3.47; 95% CI: 1.81–6.66; all estimates from Model 2).

**Table 3:** Role of individual risk factors in transitions^a^ from a healthy state to first cardiometabolic disease, multimorbidity, and mortality.

Risk factor^b^	Hazard ratio (95% CI) for transition				
	A (healthy to first disease)	B (first disease to multimorbidity)	C (healthy to mortality)	D (first disease to mortality)	E (multimorbidity to mortality)
**Behavioural factors**					
Physically inactive^**c**^	1.08 (1.00 1.17)	1.14 (0.95, 1.37)	1.08 (0.94, 1.24)	1.14 (0.92, 1.40)	1.07 (0.76, 1.51)
Poor diet^d^	1.09 (1.00, 1.18)	1.14 (0.96, 1.36)	1.31 (1.15, 1.50)	1.17 (0.95, 1.44)	1.79 (1.29, 2.48)
Alcohol abstention or heavy alcohol consumption^e^	1.11 (1.03, 1.21)	1.30 (1.09, 1.56)	1.25 (1.09, 1.43)	1.17 (0.95, 1.45)	1.39 (1.00, 1.94)
Current smoker	1.43 (1.30, 1.59)	1.57 (1.28, 1.92)	2.44 (2.10, 2.84)	1.72 (1.36,2.16)	1.59 (1.12, 2.26)
**Clinical profile**					
Hypertension^f^	1.53 (1.40, 1.67)	1.02 (0.85, 1.24)	1.47 (1.26, 1.71)	1.30 (1.05, 1.61)	0.97 (0.69, 1.37)
Overweight/obesity^g^	1.70 (1.56, 1.84)	1.19 (1.00, 1.43)	1.15 (1.01, 1.31)	1.06 (0.86, 1.30)	0.85 (0.61, 1.18)
Total cholesterol ≥ 5 mmol/l	1.30 (1.14, 1.48)	1.08 (0.80, 1.46)	0.93 (0.76, 1.13)	0.87 (0.63, 1.22)	0.57 (0.35, 0.93)
Family history of diabetes or CVD	1.21 (1.09, 1.33)	0.94 (0.76, 1.18)	0.84 (0.70, 1.01)	0.98 (0.76, 1.27)	0.89 (0.58, 1.35)

^a^For transitions see Fig 1.

^b^Individual risk factors are dichotomous; reference group is composed of persons without the risk factor.

^c^Corresponds to <2.5 hours/week of moderate or vigorous physical activity (recommended level).

^d^Corresponds to fruit and vegetable consumption < 1 serving/day.

^e^Heavy alcohol consumption was defined as >14 units/week in women and >21 units/week in men, and alcohol abstention was defined as <1 unit/week.

^f^Blood pressure ≥ 140/90 mm Hg or use of antihypertensive medication.

^g^BMI ≥ 25 kg/m^2^.

CVD, cardiovascular disease.

**Table 4:** Role of occupation, behavioural factors, and clinical profile in transitions^a^ from a healthy state to first cardiometabolic disease, multimorbidity, and mortality.

Transition	*N* events/total	Hazard ratio (95% CI)^b^ for risk factor					
		Model 1			Model 2		
		Occupation	Behavioural factors	Clinical profile	Occupation	Behavioural factors	Clinical profile
A (healthy to first disease)	2,501/8,270	1.57 (1.36, 1.81)	1.59 (1.35, 1.86)	3.81 (3.20, 4.53)	1.42 (1.23, 1.64)	1.44 (1.22, 1.69)	3.74 (3.14, 4.45)
B (first disease to multimorbidity)	511/2,501	1.77 (1.27, 2.47)	2.18 (1.54, 3.09)	1.28 (0.86, 1.91)	1.54 (1.10, 2.15)	2.00 (1.40, 2.85)	1.29 (0.86, 1.94)
C (healthy to mortality)	872/8,270	1.64 (1.29, 2.08)	3.08 (2.36, 4.02)	1.47 (1.09, 1.99)	1.33 (1.04, 1.69)	2.87 (2.19, 3.77)	1.42 (1.05, 1.93)
D (first disease to mortality)	383/2,501	1.18 (0.81, 1.70)	2.09 (1.40, 3.11)	1.38 (0.87, 2.19)	1.00 (0.69, 1.45)	2.12 (1.41, 3.18)	1.42 (0.89, 2.26)
E (multimorbidity to mortality)	151/511	2.30 (1.21, 4.39)	3.82 (2.01, 7.25)	0.64 (0.31, 1.34)	1.65 (0.86, 3.20)	3.47 (1.81, 6.66)	0.67 (0.32, 1.40)

Model 1: analysis adjusted for age, sex, race, marital status, and birth cohort. Model 2: Model 1 plus mutual adjustment for scales of occupation, behavioural factors, and clinical profile.

^a^For transitions see Fig 1.

^b^Hazard ratio for lowest versus highest in scales of occupation, behavioural factors, and clinical profile.

In sensitivity analyses, we examined the role of risk factor scales in transitions separately in men and women (S5 Table). These results show small differences in the role of occupational position but no differences for behavioural or clinical risk factor scales. In further analyses, we used education rather than occupation as a marker of socioeconomic circumstances (S6 Table); these results were broadly consistent with our main findings.

## Discussion

The emerging body of research on multimorbidity has assessed the role of risk factors for multimorbidity [3,6,8,16–20] or examined the risk of mortality in those with multimorbidity [4]. In contrast, we examined the role of clinical, socioeconomic, and behavioural risk factors at age 50 years in modifying transitions in health using MSMs, an approach that allows the assessment of both etiological and prognostic factors within a single analytic framework. We present 3 key findings. One, clinical parameters were the strongest predictors of the incidence of a first cardiometabolic disease, but they played a more modest role in progression from a single disease to cardiometabolic multimorbidity or risk of mortality in those with cardiometabolic multimorbidity. Two, midlife socioeconomic and behavioural factors were found to be important predictors of multimorbidity in those with existing cardiometabolic disease. Three, midlife behavioural factors were important predictors of mortality in those free of cardiometabolic diseases, those with 1 cardiometabolic disease, and those with cardiometabolic multimorbidity: the respective HRs for those with the worst compared to the best behavioural profile all exceed 2, indicating their considerable impact on mortality risk.

The objective of our analysis was to identify how known clinical, socioeconomic, and behavioural risk factors shape the course of cardiometabolic disease. The precise pathophysiological mechanisms underlying the association of risk factors with disease incidence and prognosis were not examined. Each of our 3 main findings has implications for prevention and care of persons with cardiometabolic disease. The importance of clinical parameters, individually and when considered as a scale for their combined impact, for the incidence of a first cardiometabolic disease suggests that basic clinical factors are not yet being targeted sufficiently in primary prevention. The decline in deaths from cardiovascular disease in high-income countries is attributed in large part to such prevention strategies [28,29]. However, our data show large variation in clinical factors and that an adverse clinical profile is associated with a 3.7-fold greater hazard of a first cardiometabolic disease in fully adjusted analyses. These findings should encourage further efforts to promote lower body weight, blood pressure, and total cholesterol.

Our second finding highlights the role of socioeconomic and behavioural factors as being important predictors of multimorbidity in those with pre-existing cardiometabolic disease. Much of the research on multimorbidity has attempted to identify risk factors for multimorbidity, but not specifically the factors that lead to multimorbidity in those with a first cardiometabolic disease. Previous studies have shown socioeconomic factors [3,11] and behavioural factors [16,19,20] to be associated with multimorbidity; however, some of these studies were cross-sectional [3,11,19,30,31] and used self-reported measures of multimorbidity [19]. No previous study to our knowledge has examined the role of risk factors in the progression from 1 disease to multimorbidity and death within a single analytic framework. The conventional approach, demonstrated in our analyses using Cox regression (Table 2), suggests that the clustering of clinical risk factors is important both for a first cardiometabolic disease and cardiometabolic multimorbidity. This is not the case when the role of risk factors was examined in the risk of multimorbidity in those with 1 cardiometabolic disease, where clinical factors were not associated with multimorbidity but both behavioural and socioeconomic risk scales predicted progression to multimorbidity, highlighting the need to understand the modifiers of disease progression for effective prevention. Socioeconomic factors have long been known to be major determinants of health [21], and continue to be seen to be important compared to traditional risk factors [32]. Our results show them to be important predictors of multimorbidity in persons with a first cardiometabolic condition even when the effects of clinical and behavioural factors have been taken into account in the analysis.

The third finding highlights the key role of adverse midlife behavioural factors in risk of mortality, in those without or with 1 or 2 or more cardiometabolic diseases. In those with cardiometabolic multimorbidity, adverse behavioural factors were associated with as high as a 3.5-fold increased risk of death. Among the individual behavioural risk factors, our results highlight the role of smoking in all 5 transitions from a healthy state to mortality. Previous research has examined the risk of mortality in those with multimorbidity compared to disease-free individuals; a recent paper found each of 3 cardiometabolic conditions to be associated with a similar increased risk of mortality, and a combination of these diseases was associated with a multiplicative mortality risk [4]. The multimorbidity–mortality association depends on the specific chronic conditions used to characterise multimorbidity, whether they were self-reported, and the duration of follow-up [11]. It is worth noting that few studies have examined factors that modify the association of multimorbidity with mortality. There is some research on interventions, mainly on the organisation of care delivery in multimorbid patients, but the findings are inconclusive [12]. Our study is the first to our knowledge to examine the relative importance of clinical, socioeconomic, and behavioural factors in modifying disease trajectories; these findings highlight the major role of behavioural factors. Previous findings have highlighted the role of behavioural factors in mortality [33,34]; our findings show them to also be critically important for secondary prevention.

The ageing of populations and reduced case fatality of major chronic conditions has led to rapid increases in the prevalence of multimorbidity [3,10,11,30,31]; Scottish data on 1.7 million adults registered with primary care providers show the prevalence of multimorbidity to increase from 30% in those aged 45–64 to 65% in those aged 65–84 years [8]. However, the definition of multimorbidity varies across studies. A simple count of all chronic conditions yields prevalence rates of between 17% and 98%, depending on the conditions included in the list [35]. A recent study used up to 40 conditions to define multimorbidity, and reported 42% of persons to have 1 disease and 23% to have 2 or more diseases [8]. In our data, 30% had 1 cardiometabolic disease, and 6% had cardiometabolic multimorbidity. Our use of a narrow set of conditions that are leading causes of death or burden of disease allows the identification of common etiopathogenic and prognostic factors. Better understanding of these patterns may lead to improvements in preventive actions to reduce prevalence and may also give rise to new, more comprehensive approaches to the management of multimorbidity.

Our findings need to be considered in light of the study strengths and limitations. The strengths of our study derive from the large sample size, the long follow-up to allow analyses on clinically diagnosed incident diseases, and the availability of complete data on health outcomes. Although a gold-standard definition of multimorbidity remains elusive, there is consensus on use of clinically assessed rather than self-reported conditions [36]. A further strength is our analytic strategy. To illustrate its value, we also report results of conventional survival analyses (Table 2), which show clinical parameters to be important predictors of cardiometabolic multimorbidity. However, they are less important in shaping temporal disease progression, as clinical parameters were not associated with progression to multimorbidity in those with first cardiometabolic disease or mortality in those with cardiometabolic multimorbidity. Thus, the large body of research on risk and prognostic factors on individual disease outcomes may not be transferable to multimorbidity. We chose to study the role of risk factors at age 50 as risk factors assessed at older ages, in particular health-related behaviours, are likely to be prone to reverse causation biases, and, for clinical risk factors, there is evidence of significant tracking effects, defined as the longitudinal stability of the predictability of risk factors over time [37].

The primary limitation of our study is uncertain generalisability of findings as the results from an occupational cohort from the UK such as the Whitehall II study are likely to apply only to high-income countries with universal healthcare. A further limitation is that study participants in occupational cohorts tend to be healthier than individuals in the general population; however, this is an unlikely source of bias in risk factor–disease associations as we have shown estimates from our study to be similar to those reported in general-population-based studies [38]. As the Whitehall II study was designed to include a wide **s**ocioeconomic spectrum, with a 10-fold salary difference among the participants, the findings on socioeconomic factors are likely to be generalisable [32]. Temporal changes in risk factor distributions over the course of the study are unlikely to affect generalisability [39] as we estimated risk factor–outcome associations rather than composite measures such as population attributable fractions that also incorporate information on prevalence of risk factors. All risk factors were assessed at age 50; changes in risk factor levels due to treatment or lifestyle modification were not examined in our study. As the data come from an observational study, the findings may be affected by unobserved confounders. Finally, we chose to model 5 transitions rather than all possible transitions between individual diseases and outcomes and between pairs of diseases in order to simplify the analysis and interpretation of results. Transition A (healthy to first cardiometabolic disease) is comparable to analysis of incident cardiometabolic disease using Cox regression; similar results from both approaches suggest that our estimates of the transitions examined are robust and fit the data well.

In conclusion, multimorbidity is increasingly a challenge for patients, healthcare providers, and the healthcare systems globally, but medical research and healthcare delivery continue to focus on individual diseases. Our analysis of the natural history of cardiometabolic multimorbidity shows clinical parameters to be key determinants of incidence of a first cardiometabolic disease but socioeconomic and behavioural factors to determine progression to multimorbidity and behavioural factors to be important in shaping risk of mortality. These results highlight the need for a comprehensive approach to the primary and secondary prevention of cardiometabolic diseases.

## Supporting information

S1 Checklist STROBE Statement for observational studies. (DOCX)

S1 Fig Flowchart of sample selection. (TIF)

S1 Protocol Prospective analysis plan. (DOCX)

S1 Table Characteristics of the study population (total *N* = 8,270) at age 50 as a function of cardiometabolic disease status at the end of follow-up. (DOCX)

S2 Table Association of time-varying cardiometabolic disease and multimorbidity with mortality. (DOCX)

S3 Table Association of individual risk factors with cardiometabolic disease and multimorbidity using Cox regression (total N = 8,270). (DOCX)

S4 Table Cox regression to assess associations of occupation, behavioural factors, and clinical profile with first cardiometabolic disease, multimorbidity, and mortality in mutually adjusted models. (DOCX)

S5 Table Role of occupation, behavioural factors, and clinical profile in transitions from a healthy state to first cardiometabolic disease, multimorbidity, and mortality in men and women. (DOCX)

S6 Table Role of education, behavioural factors, and clinical profile in transitions from a healthy state to first cardiometabolic disease, cardiometabolic multimorbidity, and mortality. (DOCX)

## Abbreviations

CHD: coronary heart disease
HES: Hospital Episode Statistics
HR: hazard ratio
MSM: multi-state model
NHS: National Health Service

## References

[1] Bayliss EA, Edwards AE, Steiner JF, Main DS. Processes of care desired by elderly patients with multimorbidities. Fam Pract. 2008;25(4):287–93. doi: 10.1093/fampra/cmn040 18628243 10.1093/fampra/cmn040 PMC2504745

[2] Agborsangaya CB, Lau D, Lahtinen M, Cooke T, Johnson JA. Multimorbidity prevalence and patterns across socioeconomic determinants: a cross-sectional survey. BMC Public Health. 2012;12:201 doi: 10.1186/1471-2458-12-201 22429338 10.1186/1471-2458-12-201 PMC3353224

[3] McLean G, Gunn J, Wyke S, Guthrie B, Watt GC, Blane DN, et al The influence of socioeconomic deprivation on multimorbidity at different ages: a cross-sectional study. Br J Gen Pract. 2014;64(624):e440–7. doi: 10.3399/bjgp14X680545 24982497 10.3399/bjgp14X680545 PMC4073730

[4] Emerging Risk Factors Collaboration, Di Angelantonio E, Kaptoge S, Wormser D, Willeit P, Butterworth AS, et al Association of cardiometabolic multimorbidity with mortality. JAMA. 2015;314(1):52–60. doi: 10.1001/jama.2015.7008 26151266 10.1001/jama.2015.7008 PMC4664176

[5] Weiss CO, Boyd CM, Yu Q, Wolff JL, Leff B. Patterns of prevalent major chronic disease among older adults in the United States. JAMA. 2007;298(10):1160–2. doi: 10.1001/jama.298.10.1160-b 17848649 10.1001/jama.298.10.1160-b

[6] Tucker-Seeley RD, Li Y, Sorensen G, Subramanian SV. Lifecourse socioeconomic circumstances and multimorbidity among older adults. BMC Public Health. 2011;11:313 doi: 10.1186/1471-2458-11-313 21569558 10.1186/1471-2458-11-313 PMC3118239

[7] St Sauver JL, Boyd CM, Grossardt BR, Bobo WV, Finney Rutten LJ, Roger VL, et al Risk of developing multimorbidity across all ages in an historical cohort study: differences by sex and ethnicity. BMJ Open. 2015;5(2):e006413 doi: 10.1136/bmjopen-2014-006413 25649210 10.1136/bmjopen-2014-006413 PMC4322195

[8] Barnett K, Mercer SW, Norbury M, Watt G, Wyke S, Guthrie B. Epidemiology of multimorbidity and implications for health care, research, and medical education: a cross-sectional study. Lancet. 2012;380(9836):37–43. doi: 10.1016/S0140-6736(12)60240-2 22579043 10.1016/S0140-6736(12)60240-2

[9] Tinetti ME, Fried TR, Boyd CM. Designing health care for the most common chronic condition—multimorbidity. JAMA. 2012;307(23):2493–4. doi: 10.1001/jama.2012.5265 22797447 10.1001/jama.2012.5265 PMC4083627

[10] Vogeli C, Shields AE, Lee TA, Gibson TB, Marder WD, Weiss KB, et al Multiple chronic conditions: prevalence, health consequences, and implications for quality, care management, and costs. J Gen Intern Med. 2007;22(Suppl 3):391–5. doi: 10.1007/s11606-007-0322-1 18026807 10.1007/s11606-007-0322-1 PMC2150598

[11] Marengoni A, Angleman S, Melis R, Mangialasche F, Karp A, Garmen A, et al Aging with multimorbidity: a systematic review of the literature. Ageing Res Rev. 2011;10(4):430–9. doi: 10.1016/j.arr.2011.03.003 21402176 10.1016/j.arr.2011.03.003

[12] Smith SM, Soubhi H, Fortin M, Hudon C, O’Dowd T. Managing patients with multimorbidity: systematic review of interventions in primary care and community settings. BMJ. 2012;345:e5205 doi: 10.1136/bmj.e5205 22945950 10.1136/bmj.e5205 PMC3432635

[13] Beard JR, Officer A, de Carvalho IA, Sadana R, Pot AM, Michel JP, et al The world report on ageing and health: a policy framework for healthy ageing. Lancet. 2016;387(10033):2145–54. doi: 10.1016/S0140-6736(15)00516-4 26520231 10.1016/S0140-6736(15)00516-4 PMC4848186

[14] Valderas JM, Starfield B, Sibbald B, Salisbury C, Roland M. Defining comorbidity: implications for understanding health and health services. Ann Fam Med. 2009;7(4):357–63. doi: 10.1370/afm.983 19597174 10.1370/afm.983 PMC2713155

[15] Wolff JL, Starfield B, Anderson G. Prevalence, expenditures, and complications of multiple chronic conditions in the elderly. Arch Intern Med. 2002;162(20):2269–76. 12418941 10.1001/archinte.162.20.2269

[16] Wikstrom K, Lindstrom J, Harald K, Peltonen M, Laatikainen T. Clinical and lifestyle-related risk factors for incident multimorbidity: 10-year follow-up of Finnish population-based cohorts 1982–2012. Eur J Intern Med. 2015;26(3):211–6. doi: 10.1016/j.ejim.2015.02.012 25747490 10.1016/j.ejim.2015.02.012

[17] Kivimaki M, Kuosma E, Ferrie JE, Luukkonen R, Nyberg ST, Alfredsson L, et al Overweight, obesity, and risk of cardiometabolic multimorbidity: pooled analysis of individual-level data for 120 813 adults from 16 cohort studies from the USA and Europe. Lancet Public Health. 2017;2(6):e277–85. doi: 10.1016/S2468-2667(17)30074-9 28626830 10.1016/S2468-2667(17)30074-9 PMC5463032

[18] Nagel G, Peter R, Braig S, Hermann S, Rohrmann S, Linseisen J. The impact of education on risk factors and the occurrence of multimorbidity in the EPIC-Heidelberg cohort. BMC Public Health. 2008;8:384 doi: 10.1186/1471-2458-8-384 19014444 10.1186/1471-2458-8-384 PMC2614432

[19] Fortin M, Haggerty J, Almirall J, Bouhali T, Sasseville M, Lemieux M. Lifestyle factors and multimorbidity: a cross sectional study. BMC Public Health. 2014;14:686 doi: 10.1186/1471-2458-14-686 24996220 10.1186/1471-2458-14-686 PMC4096542

[20] Dhalwani NN, O’Donovan G, Zaccardi F, Hamer M, Yates T, Davies M, et al Long terms trends of multimorbidity and association with physical activity in older English population. Int J Behav Nutr Phys Act. 2016;13:8 doi: 10.1186/s12966-016-0330-9 26785753 10.1186/s12966-016-0330-9 PMC4717631

[21] Marmot MG, Smith GD, Stansfeld S, Patel C, North F, Head J, et al Health inequalities among British civil servants: the Whitehall II study. Lancet. 1991;337(8754):1387–93. 1674771 10.1016/0140-6736(91)93068-k

[22] Sabia S, Singh-Manoux A, Hagger-Johnson G, Cambois E, Brunner EJ, Kivimaki M. Influence of individual and combined healthy behaviours on successful aging. CMAJ. 2012;184(18):1985–92. doi: 10.1503/cmaj.121080 23091184 10.1503/cmaj.121080 PMC3519184

[23] Sabia S, Dugravot A, Dartigues JF, Abell J, Elbaz A, Kivimaki M, et al Physical activity, cognitive decline, and risk of dementia: 28 year follow-up of Whitehall II cohort study. BMJ. 2017;357:j2709 doi: 10.1136/bmj.j2709 28642251 10.1136/bmj.j2709 PMC5480222

[24] Doyle Y, Bull A. Role of private sector in United Kingdom healthcare system. BMJ. 2000;321(7260):563–5. 10968825 10.1136/bmj.321.7260.563 PMC1118448

[25] Britton A, Milne B, Butler T, Sanchez-Galvez A, Shipley M, Rudd A, et al Validating self-reported strokes in a longitudinal UK cohort study (Whitehall II): extracting information from hospital medical records versus the Hospital Episode Statistics database. BMC Med Res Methodol. 2012;12:83 doi: 10.1186/1471-2288-12-83 22720999 10.1186/1471-2288-12-83 PMC3407010

[26] Putter H, Fiocco M, Geskus RB. Tutorial in biostatistics: competing risks and multi-state models. Stat Med. 2007;26(11):2389–430. doi: 10.1002/sim.2712 17031868 10.1002/sim.2712

[27] de Wreede LC, Fiocco M, Putter H. The mstate package for estimation and prediction in non- and semi-parametric multi-state and competing risks models. Comput Methods Programs Biomed. 2010;99(3):261–74. doi: 10.1016/j.cmpb.2010.01.001 20227129 10.1016/j.cmpb.2010.01.001

[28] Unal B, Critchley JA, Capewell S. Modelling the decline in coronary heart disease deaths in England and Wales, 1981–2000: comparing contributions from primary prevention and secondary prevention. BMJ. 2005;331(7517):614 doi: 10.1136/bmj.38561.633345.8F 16107431 10.1136/bmj.38561.633345.8F PMC1215556

[29] Ford ES, Ajani UA, Croft JB, Critchley JA, Labarthe DR, Kottke TE, et al Explaining the decrease in U.S. deaths from coronary disease, 1980–2000. N Engl J Med. 2007;356(23):2388–98. doi: 10.1056/NEJMsa053935 17554120 10.1056/NEJMsa053935

[30] Afshar S, Roderick PJ, Kowal P, Dimitrov BD, Hill AG. Multimorbidity and the inequalities of global ageing: a cross-sectional study of 28 countries using the World Health Surveys. BMC Public Health. 2015;15:776 doi: 10.1186/s12889-015-2008-7 26268536 10.1186/s12889-015-2008-7 PMC4534141

[31] Garin N, Koyanagi A, Chatterji S, Tyrovolas S, Olaya B, Leonardi M, et al Global multimorbidity patterns: a cross-sectional, population-based, multi-country study. J Gerontol A Biol Sci Med Sci. 2016;71(2):205–14. doi: 10.1093/gerona/glv128 26419978 10.1093/gerona/glv128 PMC5864156

[32] Stringhini S, Carmeli C, Jokela M, Avendano M, Muennig P, Guida F, et al Socioeconomic status and the 25 x 25 risk factors as determinants of premature mortality: a multicohort study and meta-analysis of 1.7 million men and women. Lancet. 2017;389(10075):1229–37. doi: 10.1016/S0140-6736(16)32380-7 28159391 10.1016/S0140-6736(16)32380-7 PMC5368415

[33] Khaw KT, Wareham N, Bingham S, Welch A, Luben R, Day N. Combined impact of health behaviours and mortality in men and women: the EPIC-Norfolk prospective population study. PLoS Med. 2008;5(1):e12 doi: 10.1371/journal.pmed.0050012 18184033 10.1371/journal.pmed.0050012 PMC2174962

[34] Stringhini S, Sabia S, Shipley M, Brunner E, Nabi H, Kivimaki M, et al Association of socioeconomic position with health behaviors and mortality. JAMA. 2010;303(12):1159–66. doi: 10.1001/jama.2010.297 20332401 10.1001/jama.2010.297 PMC2918905

[35] Ryan A, Wallace E, O’Hara P, Smith SM. Multimorbidity and functional decline in community-dwelling adults: a systematic review. Health Qual Life Outcomes. 2015;13:168 doi: 10.1186/s12955-015-0355-9 26467295 10.1186/s12955-015-0355-9 PMC4606907

[36] Fortin M, Stewart M, Poitras ME, Almirall J, Maddocks H. A systematic review of prevalence studies on multimorbidity: toward a more uniform methodology. Ann Fam Med. 2012;10(2):142–51. doi: 10.1370/afm.1337 22412006 10.1370/afm.1337 PMC3315131

[37] Ulmer H, Kelleher C, Diem G, Concin H. Long-term tracking of cardiovascular risk factors among men and women in a large population-based health system: the Vorarlberg Health Monitoring & Promotion Programme. Eur Heart J. 2003;24(11):1004–13. 12788300 10.1016/s0195-668x(03)00170-2

[38] Batty GD, Shipley M, Tabak A, Singh-Manoux A, Brunner E, Britton A, et al Generalizability of occupational cohort study findings. Epidemiology. 2014;25(6):932–3. doi: 10.1097/EDE.0000000000000184 25265141 10.1097/EDE.0000000000000184

[39] Navar AM, Peterson ED, Wojdyla D, Sanchez RJ, Sniderman AD, D’Agostino RB Sr, et al Temporal changes in the association between modifiable risk factors and coronary heart disease incidence. JAMA. 2016;316(19):2041–3. doi: 10.1001/jama.2016.13614 27838711 10.1001/jama.2016.13614 PMC5547567

