# Supplementary material for: Clinical, socioeconomic, and behavioural factors at age 50 years and risk of cardiometabolic multimorbidity and mortality: A cohort study
Source: PLoS Med. 2018 May 21;15(5):e1002571. doi: 10.1371/journal.pmed.1002571 (PMC5962054; doi:10.1371/journal.pmed.1002571)
Supplement: S1 Checklist — (DOCX) [file pmed.1002571.s001.docx]

**S1 Checklist. STROBE Statement for observational studies**

|  | | Item No | Recommendation |
| --- | --- | --- | --- |
| **Title and abstract** | | 1 | 1. Indicate the study’s design with a commonly used term in the title or the abstract   **This study used a longitudinal cohort as indicated in the title.**  **“Clinical, socioeconomic and behavioural factors at age 50 and risk of cardiometabolic multimorbidity and mortality: a cohort study”** |
|  |  |  | 1. Provide in the abstract an informative and balanced summary of what was done and what was found   **The abstract contains the following sections: background, methods & findings, and conclusions.** |
| Introduction | | | |
| Background/rationale | | 2 | Explain the scientific background and rationale for the investigation being reported  **This is outlined in paragraphs 1 & 3 of the introduction.** |
| Objectives | | 3 | State specific objectives, including any prespecified hypotheses  **This is outlined in paragraphs 3 of the introduction.** |
| Methods | | | |
| Study design | | 4 | Present key elements of study design early in the paper  **The abstract contains all the key elements of the paper.** |
| Setting | | 5 | Describe the setting, locations, and relevant dates, including periods of recruitment, exposure, follow-up, and data collection  **This information is provided in the Methods section entitled “Study Population”.** |
| Participants | | 6 | (*a*) *Cohort study*—Give the eligibility criteria, and the sources and methods of selection of participants. Describe methods of follow-up  **This information is provided in the Methods section entitled “Study Population”** |
|  |  |  |  |
| Variables | | 7 | Clearly define all outcomes, exposures, predictors, potential confounders, and effect modifiers. Give diagnostic criteria, if applicable  **This information is described in the methods section under the headings:**  **Assessment of risk factors**  **Ascertainment of cardiometabolic diseases and multimorbidity**  **Mortality follow-up**  **Assessment of sex differences in Table 2 and S5 Table.** |
| Data sources/ measurement | | 8* | For each variable of interest, give sources of data and details of methods of assessment (measurement). Describe comparability of assessment methods if there is more than one group  **This information is described in the methods section under the heading**  **Assessment of risk factors** |
| Bias | | 9 | Describe any efforts to address potential sources of bias  **We compare Cox regression with multi-state models, the latter takes into account competing risk due to mortality.** |
| Study size | | 10 | Explain how the study size was arrived at  **S1 Figure(flow chart) provides details of how we arrived at the sample size.** |
| Quantitative variables | | 11 | Explain how quantitative variables were handled in the analyses. If applicable, describe which groupings were chosen and why  **This is described in the Methods section.** |
| Statistical methods | |  | (*a*) Describe all statistical methods, including those used to control for confounding **The statistical methods are described on page 10-11.** |
|  |  |  | 1. Describe any methods used to examine subgroups and interactions   **Assessment of sex differences in Table 2 and S5 Table.** |
|  |  |  | 1. Explain how missing data were addressed   **No missing data due to complete linkage to records.** |
|  |  |  | 1. *Cohort study*—If applicable, explain how loss to follow-up was addressed   **No loss to follow-up.** |
|  |  |  | 1. Describe any sensitivity analyses   **Sensitivity analyses: education replaced occupation as a marker of socioeconomic position (S6 Table)** |
| Results | | | |
| Participants | 13* | 1. Report numbers of individuals at each stage of study—eg numbers potentially eligible, examined for eligibility, confirmed eligible, included in the study, completing follow-up, and analysed   **S1 Figure provides details on the composition of the analytic sample.** | |
|  |  | 1. Give reasons for non-participation at each stage   **Results section, paragraph 1.** | |
|  |  | 1. Consider use of a flow diagram   **A flow diagram is included as S1 Figure.** | |
| Descriptive data | 14* | 1. Give characteristics of study participants (eg demographic, clinical, social) and information on exposures and potential confounders   **These characteristics are included in Table 1 & S1 Table.** | |
|  |  | 1. Indicate number of participants with missing data for each variable of interest   **These data are provided in S1 Figure.** | |
|  |  | 1. *Cohort study*—Summarise follow-up time (eg, average and total amount)   **The results section and tables provide these data.** | |
| Outcome data | 15* | *Cohort study*—Report numbers of outcome events or summary measures over time  **The outcome numbers are described in the abstract, results section and Tables.** | |
|  |  | *Case-control study—*Report numbers in each exposure category, or summary measures of exposure  **Not relevant.** | |
|  |  | *Cross-sectional study—*Report numbers of outcome events or summary measures  **Not relevant.** | |
| Main results | 16 | 1. Give unadjusted estimates and, if applicable, confounder-adjusted estimates and their precision (eg, 95% confidence interval). Make clear which confounders were adjusted for and why they were included   **Tables 2 and 3.** | |
|  |  | (*b*) Report category boundaries when continuous variables were categorized  **Not relevant** | |
|  |  | 1. If relevant, consider translating estimates of relative risk into absolute risk for a meaningful time period   **Not relevant.** | |
| Other analyses | 17 | Report other analyses done—eg analyses of subgroups and interactions, and sensitivity analyses  **The** **Supplementary data provide details of analyses undertaken to complement those reported in the manuscript.** | |
| Discussion | | | |
| Key results | 18 | Summarise key results with reference to study objectives  **The first paragraph of the discussion section summarises results in relation to study objectives.** | |
| Limitations | 19 | Discuss limitations of the study, taking into account sources of potential bias or imprecision. Discuss both direction and magnitude of any potential bias  **Limitations of the study are discussed in the penultimate paragraph of the discussion section.** | |
| Interpretation | 20 | Give a cautious overall interpretation of results considering objectives, limitations, multiplicity of analyses, results from similar studies, and other relevant evidence  **The discussion section considers our results in light of previous findings in this domain.** | |
| Generalisability | 21 | Discuss the generalisability (external validity) of the study results  **This is described in the discussion section, penultimate paragraph.** | |
| Other information | | | |
| Funding | 22 | Give the source of funding and the role of the funders for the present study and, if applicable, for the original study on which the present article is based  **Detailed in the additional submission required information, as follows.**  **This project has received funding from the European Union’s Horizon 2020 research and innovation action under grant agreements No 643576 (FRESHER) and EC 633666 (LIFEPATH). The Whitehall II study is supported by grants from the US National Institutes on Aging (R56AG056477; R01AG034454) and the UK Medical Research Council (MRC, K013351 and R024227). MK is supported by the MRC, the Academy of Finland (331492), and NordForsk.** | |

*Give information separately for cases and controls in case-control studies and, if applicable, for exposed and unexposed groups in cohort and cross-sectional studies.

**Note:** An Explanation and Elaboration article discusses each checklist item and gives methodological background and published examples of transparent reporting. The STROBE checklist is best used in conjunction with this article (freely available on the Web sites of PLoS Medicine at http://www.plosmedicine.org/, Annals of Internal Medicine at http://www.annals.org/, and Epidemiology at http://www.epidem.com/). Information on the STROBE Initiative is available at www.strobe-statement.org.
