# Supplementary material for: Clinical, socioeconomic, and behavioural factors at age 50 years and risk of cardiometabolic multimorbidity and mortality: A cohort study
Source: PLoS Med. 2018 May 21;15(5):e1002571. doi: 10.1371/journal.pmed.1002571 (PMC5962054; doi:10.1371/journal.pmed.1002571)
Supplement: S1 Protocol — (DOCX) [file pmed.1002571.s003.docx]

**S1 Protocol. Prospective analysis plan**

The Whitehall II Study was established in 1985 to study social determinants in health and has involved seven clinical assessments so far.

**Data analysis for the current project**

The analysis undertaken in this manuscript is described in our NIH funded project, EDUCATION AND AGING: TRANSITIONS FROM MULTI-MORBIDITY TO FUNCTIONAL LIMITATIONS; R56AG056477.

The abstract from this project is as follows; <https://projectreporter.nih.gov/project_info_description.cfm?aid=9565689>

**Please see below an extract from the grant with a description of the transitions examined in the paper.**

**AIM 2.** Examine whether education (academic qualification and literacy) buffers the transitions from one chronic disease to multimorbidity and to subsequent mortality using multi-state-models and identify underlying mechanisms.

1.
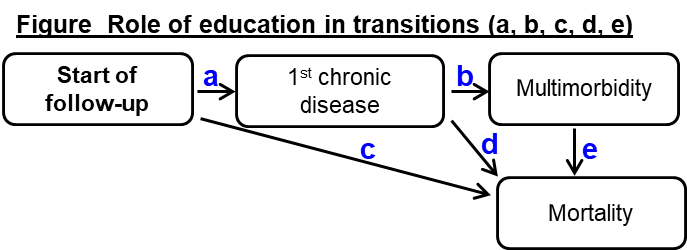
Examine the role of education in transitions from first chronic disease to multimorbidity and mortality (transitions **a** to **e** in the figure)
2. Examine whether socioeconomic, psychosocial, behavioral and biological factors are equally important across transitions from a first chronic disease to mortality (transitions **a** to **e** in the figure).
