# Supplementary material for: Clinical, socioeconomic, and behavioural factors at age 50 years and risk of cardiometabolic multimorbidity and mortality: A cohort study
Source: PLoS Med. 2018 May 21;15(5):e1002571. doi: 10.1371/journal.pmed.1002571 (PMC5962054; doi:10.1371/journal.pmed.1002571)
Supplement: S1 Table — (DOCX) [file pmed.1002571.s004.docx]

**S1 Table. Characteristics of the study population (N = 8270) at age 50 as a function of cardiometabolic disease status at the end of follow-up.**

|  | **Diabetes** | | |  | **Coronary heart disease** | | |  |  | **Stroke** |  |
| --- | --- | --- | --- | --- | --- | --- | --- | --- | --- | --- | --- |
|  | No | Yes | p |  | No | Yes | p |  | No | Yes | p |
| N | 6984 | 1286 |  |  | 6755 | 1515 |  |  | 7945 | 325 |  |
| Age, Mean (SD) | 50.2(2.4) | 50.4(2.4) | 0.0003 |  | 50.1(2.3) | 50.8(2.4) | <0.001 |  | 50.2(2.4) | 51.0(2.4) | <0.001 |
| Male, % | 67.2 | 66.9 | 0.84 |  | 66.0 | 72.3 | <0.001 |  | 67.1 | 68.3 | 0.64 |
| Non-white, % | 8.2 | 21.3 | <0.001 |  | 9.1 | 15.3 | <0.001 |  | 10.0 | 14.8 | 0.006 |
| Single, % | 24.5 | 23.9 | 0.65 |  | 25.2 | 20.8 | <0.001 |  | 24.4 | 23.4 | 0.67 |
| No academic qualification, % | 10.2 | 12.8 | <0.001 |  | 9.9 | 13.7 | <0.001 |  | 10.4 | 14.8 | 0.008 |
| Low occupational position (clerical/support staff), % | 18.1 | 26.7 | <0.001 |  | 18.6 | 23.2 | <0.001 |  | 19.1 | 26.5 | 0.007 |
| Physically inactive,^a^ % | 49.5 | 55.4 | <0.001 |  | 50.7 | 49.2 | 0.30 |  | 50.4 | 52.3 | 0.49 |
| Poor diet,^b^ % | 36.9 | 43.6 | <0.001 |  | 37.4 | 40.1 | 0.06 |  | 37.7 | 42.8 | 0.07 |
| Abstainers/heavy alcohol consumption,^c^ % | 36.5 | 41.1 | 0.002 |  | 37.2 | 37.2 | 0.99 |  | 37.0 | 43.1 | 0.03 |
| Current smokers, % | 15.0 | 18.4 | 0.002 |  | 14.5 | 20.2 | <0.001 |  | 15.1 | 25.2 | <0.001 |
| Hypertension,^d^ % | 19.3 | 29.6 | <0.001 |  | 19.3 | 27.9 | <0.001 |  | 20.7 | 26.5 | 0.01 |
| Overweight,^e^ % | 45.1 | 66.3 | <0.001 |  | 46.7 | 56.1 | <0.001 |  | 48.6 | 44.3 | 0.13 |
| Total cholesterol ≥5 mmol/L, % | 86.3 | 90.0 | <0.001 |  | 85.9 | 91.2 | <0.001 |  | 86.9 | 85.9 | 0.58 |
| Family history of diabetes or CVD,% | 17.2 | 22.6 | <0.001 |  | 18.1 | 17.8 | 0.82 |  | 17.9 | 22.2 | 0.05 |

^a^Corresponds to <2.5h of moderate and vigorous physical activity (recommended level).

^b^Corresponds to fruit and vegetable consumption <once a day.

^c^Heavy alcohol consumption was defined as >14 units/week in women and > 21 units/week in men and abstainers defined as <1 unit/week.

^d^≥140/90 mm Hg or use of antihypertensive medication.

^e^BMI≥25 kg/m^2^.

CVD: Cardiovascular Disease
