# Supplementary material for: Clinical, socioeconomic, and behavioural factors at age 50 years and risk of cardiometabolic multimorbidity and mortality: A cohort study
Source: PLoS Med. 2018 May 21;15(5):e1002571. doi: 10.1371/journal.pmed.1002571 (PMC5962054; doi:10.1371/journal.pmed.1002571)
Supplement: S2 Table — (DOCX) [file pmed.1002571.s005.docx]

**S2 Table. Association of time-varying cardiometabolic disease and multimorbidity with mortality.**

|  | **Mortality** | |
| --- | --- | --- |
| **Time-varying covariate** | N (deaths/total) | Hazard ratio (95% CI) |
| Cardiometabolic disease |  |  |
| No | 872/5769 | 1.00 [Ref.] |
| Yes | 534/2501 | 1.81 (1.61, 2.03) |
| Cardiometabolic multimorbidity |  |  |
| No | 1255/7759 | 1.00 [Ref.] |
| Yes | 151/511 | 2.36 (1.97, 2.81) |

^a^Analysis adjusted for age, sex, ethnicity, marital status, and birth cohort.
