# Supplementary material for: Clinical, socioeconomic, and behavioural factors at age 50 years and risk of cardiometabolic multimorbidity and mortality: A cohort study
Source: PLoS Med. 2018 May 21;15(5):e1002571. doi: 10.1371/journal.pmed.1002571 (PMC5962054; doi:10.1371/journal.pmed.1002571)
Supplement: S3 Table — (DOCX) [file pmed.1002571.s006.docx]

**S3 Table. Association of individual risk factors with cardiometabolic disease and multimorbidity using Cox regression, (N Total=8270).**

| **Risk factor*** | **1^st^ cardiometabolic disease** |  | **Cardiometabolic multimorbidity** |  | **Mortality** |
| --- | --- | --- | --- | --- | --- |
|  | **N events=2501** |  | **N events=511** |  | **N events=1406** |
|  | HR (95% CI) |  | HR (95% CI) |  | HR (95% CI) |
| **Behavioural factors** |  |  |  |  |  |
| Physically inactive^a^ | 1.08 (1.00, 1.17) |  | 1.21 (1.01, 1.45) |  | 1.11 (1.00, 1.24) |
| Poor diet^b^ | 1.09 (1.00, 1.18) |  | 1.20 (1.01, 1.43) |  | 1.32 (1.18, 1.46) |
| Abstainers/heavy alcohol consumption^c^ | 1.11 (1.03, 1.21) |  | 1.34 (1.13, 1.61) |  | 1.26 (1.13, 1.41) |
| Current smokers | 1.43 (1.29, 1.59) |  | 2.01 (1.64, 2.47) |  | 2.30 (2.04, 2.58) |
| **Clinical profile** |  |  |  |  |  |
| Hypertension^d^ | 1.53 (1.40, 1.67) |  | 1.49 (1.23, 1.80) |  | 1.48 (1.31, 1.66) |
| Overweight^e^ | 1.70 (1.56, 1.84) |  | 1.79 (1.50, 2.14) |  | 1.20 (1.08, 1.34) |
| Total cholesterol ≥5 mmol/L | 1.30 (1.14, 1.48) |  | 1.32 (0.98, 1.77) |  | 0.92 (0.78, 1.08) |
| Family history of diabetes or CVD | 1.21 (1.09, 1.33) |  | 1.07 (0.86, 1.34) |  | 0.91 (0.79, 1.05) |

Analysis adjusted for age, sex, ethnicity, marital status, and birth cohort.

*Individual risk factors are dichotomous; reference group is composed of persons without the risk factor.

^a^Corresponds to <2.5h of moderate and vigorous physical activity (recommended level).

^b^Corresponds to fruit and vegetable consumption <once a day.

^c^Heavy alcohol consumption was defined as >14 units/week in women and > 21 units/week in men and abstainers defined as <1 unit/week.

^d^≥140/90 mm Hg or use of antihypertensive medication .

^e^BMI≥25kg/m^2^.

CVD: Cardiovascular Disease
