# Supplementary material for: Clinical, socioeconomic, and behavioural factors at age 50 years and risk of cardiometabolic multimorbidity and mortality: A cohort study
Source: PLoS Med. 2018 May 21;15(5):e1002571. doi: 10.1371/journal.pmed.1002571 (PMC5962054; doi:10.1371/journal.pmed.1002571)
Supplement: S4 Table — (DOCX) [file pmed.1002571.s007.docx]

**S4 Table. Cox regression to assess associations of occupation, behavioural factors, and clinical profile with 1^st^ cardiometabolic disease, multimorbidity, and mortality in mutually adjusted models.**

|  | **N total** | **N**  **Events** | **1^st^ Cardiometabolic disease** |  | **N**  **Events** | **Cardiometabolic multimorbidity** |  | **N**  **Events** | **Mortality** |
| --- | --- | --- | --- | --- | --- | --- | --- | --- | --- |
|  |  |  | HR (95% CI) |  |  | HR (95% CI) |  |  | HR (95% CI) |
| **Occupation** |  |  |  |  |  |  |  |  |  |
| 0 (high) | 1410 | 380 | Ref. |  | 53 | Ref. |  | 197 | Ref. |
| 1 | 1695 | 448 | 1.05 (0.91, 1.20) |  | 66 | 1.17 (0.81, 1.67) |  | 234 | 1.11 (0.92, 1.34) |
| 2 | 2438 | 701 | 1.16 (1.02, 1.31) |  | 152 | 1.75 (1.28, 2.40) |  | 357 | 1.12 (0.94, 1.33) |
| 3 | 1122 | 380 | 1.33 (1.15, 1.55) |  | 88 | 1.78 (1.23, 2.56) |  | 229 | 1.39 (1.13, 1.71) |
| 4 (low) | 1605 | 592 | 1.41 (1.21, 1.64) |  | 152 | 1.98 (1.37, 2.85) |  | 389 | 1.37 (1.12, 1.67) |
| **Occupation scale^a^** | 8270 | 2501 | 1.45 (1.25, 1.67) |  | 511 | 2.01 (1.44, 2.80) |  | 1406 | 1.38 (1.14, 1.67) |
| **Behavioural factors** |  |  |  |  |  |  |  |  |  |
| 0 (healthiest) | 1587 | 452 | Ref. |  | 67 | Ref. |  | 189 | Ref. |
| 1 | 3063 | 857 | 0.96 (0.86, 1.08) |  | 161 | 1.19 (0.89, 1.58) |  | 453 | 1.27 (1.07, 1.51) |
| 2 | 2433 | 772 | 1.08 (0.96, 1.22) |  | 171 | 1.50 (1.12, 2.01) |  | 460 | 1.62 (1.37, 1.93) |
| 3 | 1007 | 355 | 1.27 (1.10, 1.47) |  | 94 | 2.01 (1.45, 2.78) |  | 245 | 2.13 (1.75, 2.59) |
| 4 (unhealthiest) | 180 | 65 | 1.43 (1.10, 1.86) |  | 18 | 2.57 (1.52, 4.36) |  | 59 | 3.36 (2.50, 4.53) |
| **Behavioural scale^a^** | 8270 | 2501 | 1.45 (1.23, 1.71) |  | 511 | 2.61 (1.83, 3.74) |  | 1406 | 2.94 (2.38, 3.64) |
| **Clinical profile** |  |  |  |  |  |  |  |  |  |
| 0 (healthiest) | 490 | 91 | Ref. |  | 15 | Ref. |  | 65 | Ref. |
| 1 | 2961 | 672 | 1.15 (0.92, 1.43) |  | 124 | 1.21 (0.71, 2.06) |  | 457 | 1.03 (0.79, 1.33) |
| 2 | 3201 | 1051 | 1.77 (1.43, 2.20) |  | 221 | 1.91 (1.13, 3.23) |  | 550 | 1.14 (0.88, 1.48) |
| 3 | 1431 | 605 | 2.42 (1.94, 3.02) |  | 138 | 2.64 (1.55, 4.51) |  | 294 | 1.33 (1.01, 1.74) |
| 4 (unhealthiest) | 187 | 82 | 2.65 (1.97, 3.57) |  | 13 | 2.13 (1.01, 4.48) |  | 40 | 1.60 (1.08, 2.38) |
| **Clinical profile scale^a^** | 8270 | 2501 | 3.67 (3.08, 4.38) |  | 511 | 3.65 (2.47, 5.39) |  | 1406 | 1.61 (1.27, 2.04) |

^a^HR for highest versus lowest in the scale.

Analysis adjusted for age, sex, ethnicity, marital status, and birth cohort.
