# Supplementary material for: Clinical, socioeconomic, and behavioural factors at age 50 years and risk of cardiometabolic multimorbidity and mortality: A cohort study
Source: PLoS Med. 2018 May 21;15(5):e1002571. doi: 10.1371/journal.pmed.1002571 (PMC5962054; doi:10.1371/journal.pmed.1002571)
Supplement: S5 Table — (DOCX) [file pmed.1002571.s008.docx]

**S5 Table. Role of occupation, behavioural factors, and clinical profile in transitions from a healthy state to 1^st^ cardiometabolic disease, multimorbidity, and mortality in men and women.**

| **Transition** | **N**  **Events/Total** |  | **MEN†** |  |
| --- | --- | --- | --- | --- |
|  |  | **Occupation** | **Behavioural factors** | **Clinical profile** |
|  |  | HR**^a^** (95% CI) | HR**^a^** (95% CI) | HR**^a^** (95% CI) |
| **A (healthy to 1^st^ disease)** | 1736/5551 | 1.37 (1.15, 1.63) | 1.51 (1.24, 1.83) | 4.06 (3.29, 5.00) |
| **B (1^st^ disease to multimorbidity)** | 352/1736 | 1.78 (1.22, 2.60) | 2.16 (1.42, 3.30) | 1.33 (0.81, 2.18) |
| **C (healthy to mortality)** | 555/5551 | 1.85 (1.37, 2.50) | 2.70 (1.93, 3.79) | 1.74 (1.20, 2.54) |
| **D (1^st^ disease to mortality)** | 250/1736 | 1.29 (0.83, 2.01) | 2.17 (1.32, 3.57) | 1.54 (0.85, 2.78) |
| **E (multimorbidity to mortality)** | 98/352 | 2.25 (1.07, 4.72) | 2.59 (1.15, 5.81) | 0.70 (0.29, 1.69) |
|  | **N**  **Events/Total** |  | **WOMEN†** |  |
|  |  | **Occupation** | **Behavioural factors** | **Clinical profile** |
|  |  | HR**^a^** (95% CI) | HR**^a^** (95% CI) | HR**^a^** (95% CI) |
| **A (healthy to 1^st^ disease)** | 765/2719 | 2.41 (1.80, 3.23) | 1.86 (1.39, 2.49) | 3.13 (2.27, 4.32) |
| **B (1^st^ disease to multimorbidity)** | 159/765 | 1.67 (0.77, 3.63) | 2.25 (1.19, 4.27) | 1.13 (0.56, 2.28) |
| **C (healthy to mortality)** | 317/2719 | 1.21 (0.81, 1.82) | 3.88 (2.49, 6.06) | 1.09 (0.65, 1.82) |
| **D (1^st^ disease to mortality)** | 133/765 | 1.13 (0.55, 2.32) | 2.50 (1.26, 4.95) | 1.14 (0.54, 2.41) |
| **E (multimorbidity to mortality)** | 53/159 | 0.87 (0.19, 3.93) | 4.14 (1.27, 13.5) | 0.30 (0.08, 1.07) |

^a^Hazard ratio (HR) for highest versus lowest in the scale.

Analysis adjusted for age, ethnicity, marital status, and birth cohort.

†Interaction term for sex differences in transitions

Occupation: transition A (p=0.002), transition B (p=0.75), transition C (p=0.02), transition D (p=0.76), transition E (p=0.38).

Behavioural factors: transition A (p=0.25), transition B (p=0.91), transition C (p=0.34), transition D (p=0.59), transition E (p=0.43).

Clinical profile: transition A (p=0.16), transition B (p=0.73), transition C (p=0.08), transition D (p=0.51), transition E (p=0.60).
