# Supplementary material for: Clinical, socioeconomic, and behavioural factors at age 50 years and risk of cardiometabolic multimorbidity and mortality: A cohort study
Source: PLoS Med. 2018 May 21;15(5):e1002571. doi: 10.1371/journal.pmed.1002571 (PMC5962054; doi:10.1371/journal.pmed.1002571)
Supplement: S6 Table — (DOCX) [file pmed.1002571.s009.docx]

**S6 Table. Role of education, behavioural factors, and clinical profile in transitions from a healthy state to 1^st^ cardiometabolic disease, cardiometabolic multimorbidity, and mortality.**

| **TRANSITIONS** |  |  | **Model 1** |  |  |  | **Model 2** |  |
| --- | --- | --- | --- | --- | --- | --- | --- | --- |
|  | **N**  **Events/Total** | **Education** | **Behavioural factors** | **Clinical profile** |  | **Education** | **Behavioural factors** | **Clinical profile** |
|  |  | HR**^a^** (95% CI) | HR**^a^** (95% CI) | HR**^a^** (95% CI) |  | HR**^a^** (95% CI) | HR**^a^** (95% CI) | HR**^a^** (95% CI) |
| **A (healthy to 1^st^ disease)** | 2501/8270 | 1.52 (1.30, 1.77) | 1.59 (1.35, 1.86) | 3.81 (3.20, 4.53) |  | 1.39 (1.19, 1.63) | 1.50 (1.27, 1.76) | 3.71 (3.12, 4.42) |
| **B (1^st^ disease to multimorbidity)** | 511/2501 | 1.48 (1.04, 2.10) | 2.18 (1.54, 3.09) | 1.28 (0.86, 1.91) |  | 1.38 (0.97, 1.96) | 2.13 (1.50, 3.03) | 1.31 (0.87, 1.96) |
| **C (healthy to mortality)** | 872/8270 | 1.12 (0.86, 1.46) | 3.08 (2.36, 4.02) | 1.47 (1.09, 1.99) |  | 0.98 (0.75, 1.28) | 3.07 (2.35, 4.01) | 1.43 (1.06, 1.94) |
| **D (1^st^ disease to mortality)** | 383/2501 | 0.94 (0.63, 1.39) | 2.09 (1.40, 3.11) | 1.38 (0.87, 2.19) |  | 0.87 (0.58, 1.29) | 2.15 (1.44, 3.21) | 1.42 (0.89, 2.27) |
| **E (multimorbidity to mortality)** | 151/511 | 0.91 (0.46, 1.81) | 3.82 (2.01, 7.25) | 0.64 (0.31, 1.34) |  | 0.79 (0.40, 1.56) | 3.83 (2.00, 7.30) | 0.71 (0.34, 1.46) |

^a^HR for highest versus lowest in the scale.

Model 1: Analysis adjusted for age, sex, ethnicity, marital status, and birth cohort.

Model 2: Model 1 + mutual adjustment.
